# APC-Mutated MUC4-Positive Fibroblastoma With Cytoplasmic β-Catenin: A Novel Variant Expanding Its Immunophenotypic Spectrum

**DOI:** 10.7759/cureus.102576

**Published:** 2026-01-29

**Authors:** Amanda Onalaja-Underwood, Rana Naous

**Affiliations:** 1 Pathology, The Ohio State University Wexner Medical Center, Columbus, USA

**Keywords:** apc, beta-catenin, fibroblastoma, immunophenotype, muc4

## Abstract

MUC4-positive fibroblastoma is a newly characterized fibroblastic neoplasm, typically presenting as a bland spindle cell tumor with diffuse cytoplasmic MUC4 expression, nuclear β‑catenin positivity, and *APC* gene alterations. We report a novel case in a 34-year-old woman demonstrating diffuse cytoplasmic MUC4 expression but negative cytoplasmic β‑catenin staining, with molecular confirmation of biallelic *APC* inactivation. This finding broadens the known immunophenotypic spectrum of MUC4-positive fibroblastoma and challenges current assumptions regarding β‑catenin localization in *APC*-altered soft tissue tumors. Additional clinical, radiologic, and long-term follow-up data may help refine our understanding of the biological behavior and diagnostic boundaries of this emerging entity.

## Introduction

MUC4-positive fibroblastoma is a newly described fibroblastic neoplasm, with only a single case series reported in the literature to date [1]. Recognizing this emerging entity is clinically important due to its overlap with MUC4-positive sarcomas, particularly low-grade fibromyxoid sarcoma and sclerosing epithelioid fibrosarcoma. The defining features in the original series included hyalinized, paucicellular spindle cell tumors with diffuse cytoplasmic MUC4 expression and nuclear β-catenin accumulation. *APC* gene inactivation was identified in seven of eight tumors successfully sequenced; the remaining case, which lacked an *APC* mutation, harbored alterations in *DNMT3A* and *RUNX1*.

Nuclear β-catenin localization has historically served as an important surrogate marker for canonical Wnt pathway activation in soft tissue tumors, including desmoid-type fibromatosis [2,3].

Because MUC4-positive fibroblastoma has only recently been recognized, understanding of its biological behavior, immunophenotypic spectrum, and molecular diversity remains limited. The present case expands this spectrum by demonstrating cytoplasmic β-catenin staining despite *APC* inactivation, suggesting that β-catenin localization in this tumor may be more heterogeneous than previously appreciated.

## Case presentation

**Figure 1:**
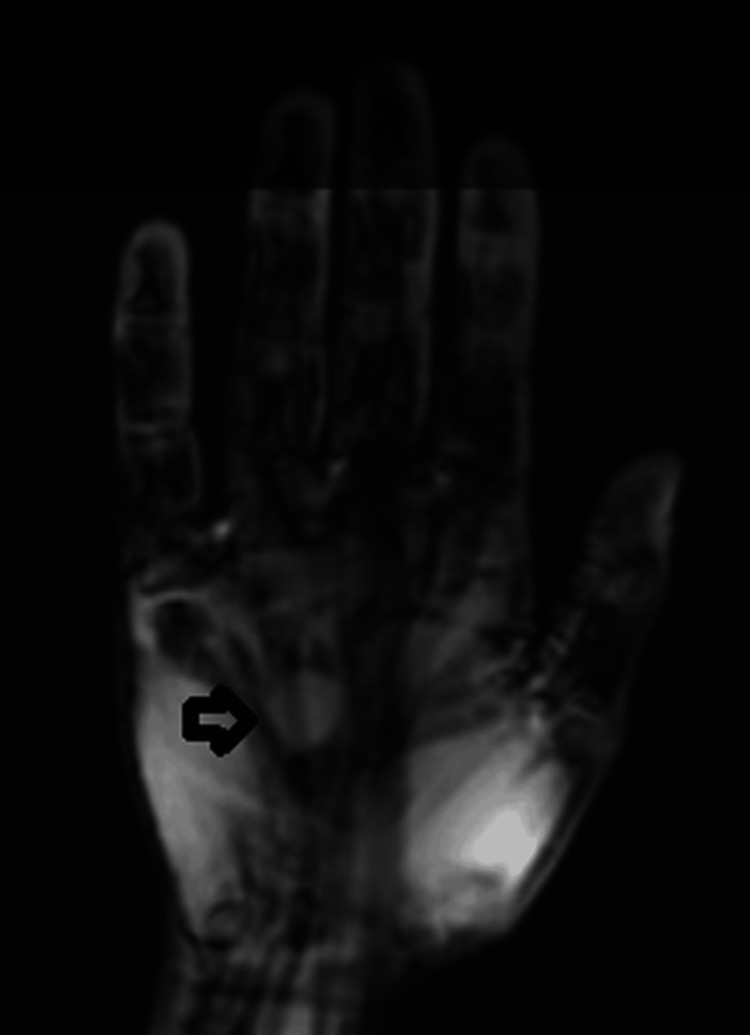
Magnetic resonance imaging of the right hand. MRI of the right hand demonstrating a well-circumscribed ovoid lesion (at arrow) centered at the level of the fourth metacarpal shaft with low signal characteristics on both T1 and T2 sequences and diffuse heterogeneous contrast enhancement.

A 33-year-old woman with a medical history of gestational diabetes and nontoxic multinodular goiter presented to her primary care physician with a 1 cm nodule on the dorsal aspect of her right hand, overlying the fourth metacarpal, which she had noticed a few days prior. She denied any history of trauma, had no prior history of cancer, and had no known familial adenomatous polyposis. Magnetic resonance imaging (Figure 1) revealed a well-circumscribed, ovoid soft tissue lesion measuring 1.3 × 1.7 × 0.7 cm, centered at the level of the fourth metacarpal shaft along the palmar surface of the hand. The lesion exhibited low signal intensity on both T1- and T2-weighted sequences, diffuse heterogeneous contrast enhancement, and no significant perilesional edema or other soft tissue abnormalities within the hand.

The lesion was closely abutting and conforming around the underlying fourth flexor digitorum superficialis tendon, with minimal contact along the peripheral margins of the third and fifth flexor digitorum superficialis tendons.

**Figure 2:**
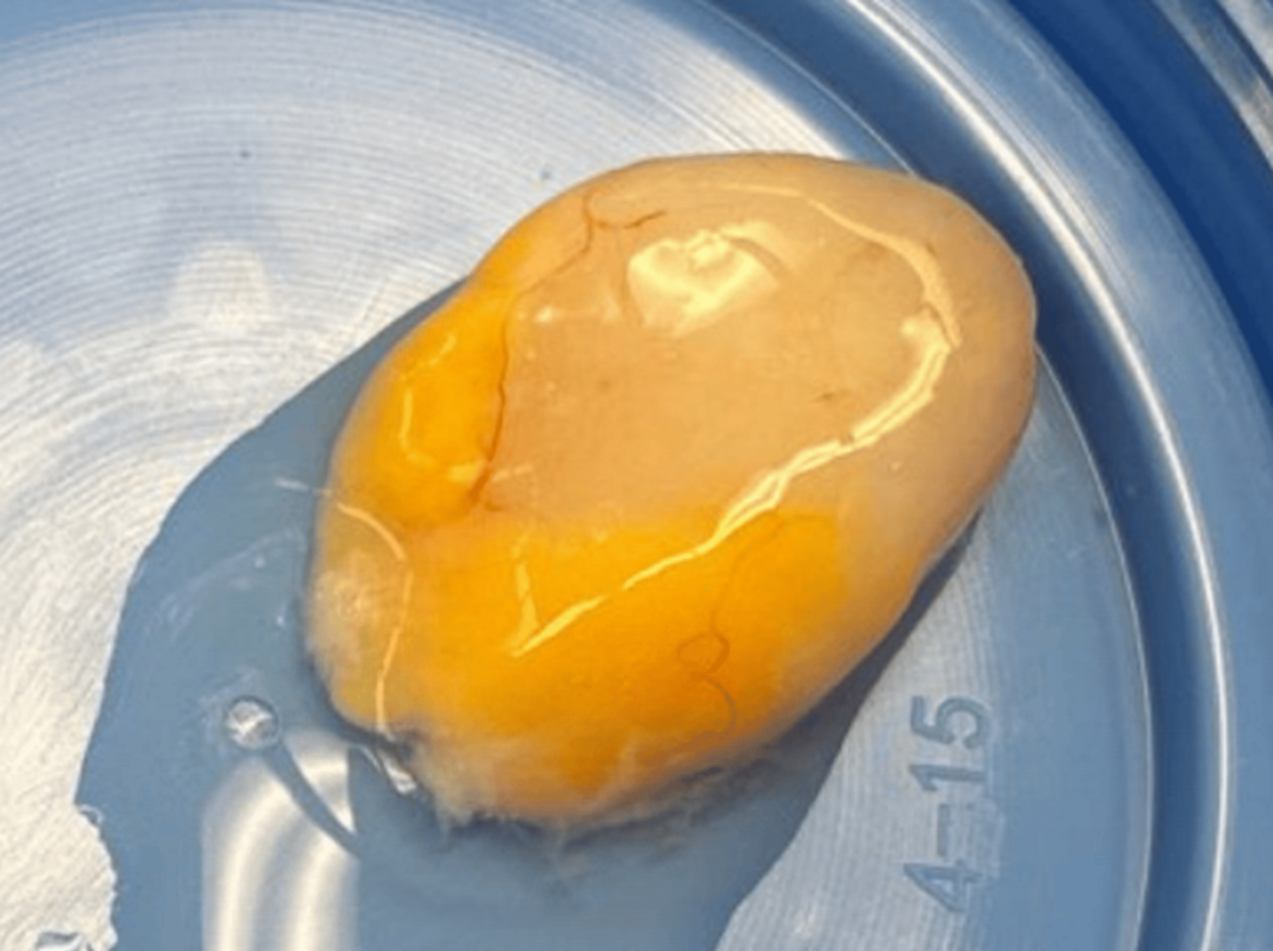
Gross image of the tumor. The tumor was firm, yellow-white, and nodular in appearance, with a glistening outer surface and measured 1.7 cm in maximum dimension.

Given its small size, surgical resection was planned. The lesion was excised without complications. Gross examination revealed a firm, yellow-white nodular mass with a glistening outer surface, measuring 1.7 cm at its greatest dimension (Figure 2).

**Figure 3:**
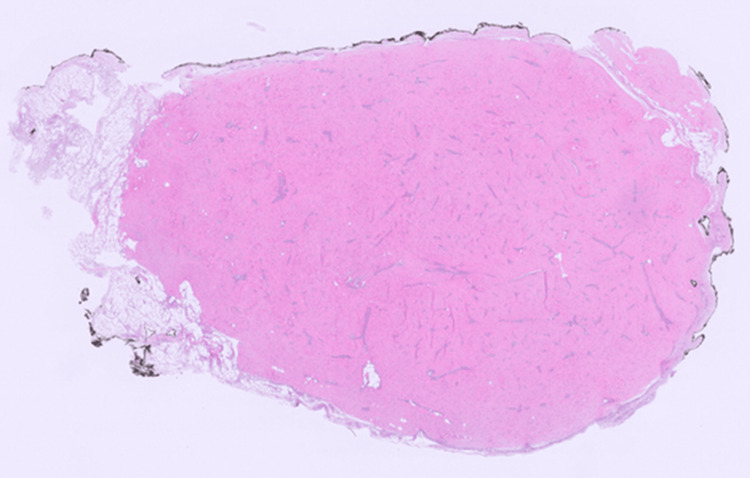
MUC-positive fibroblastoma at low magnification. On low magnification, the tumor appears relatively well-circumscribed, with mild cellularity and collagenous vascular-rich stroma (H&E, 0.25x).

Microscopically, the tumor was relatively well-circumscribed and mildly cellular, composed of bland spindle cells arranged haphazardly within a collagenous stroma with prominent vascularity (Figure 3).

**Figure 4:**
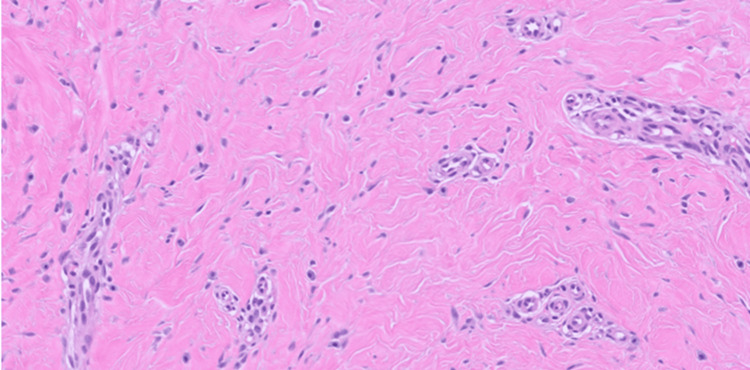
MUC4-positive fibroblastoma. The lesional cells are bland spindled with haphazard arrangement and set in a dense collagenous stroma with increased vascularity (H&E, 20x).

The lesional cells exhibited bland, elongated nuclei with scant eosinophilic cytoplasm (Figure 4).

**Figure 5:**
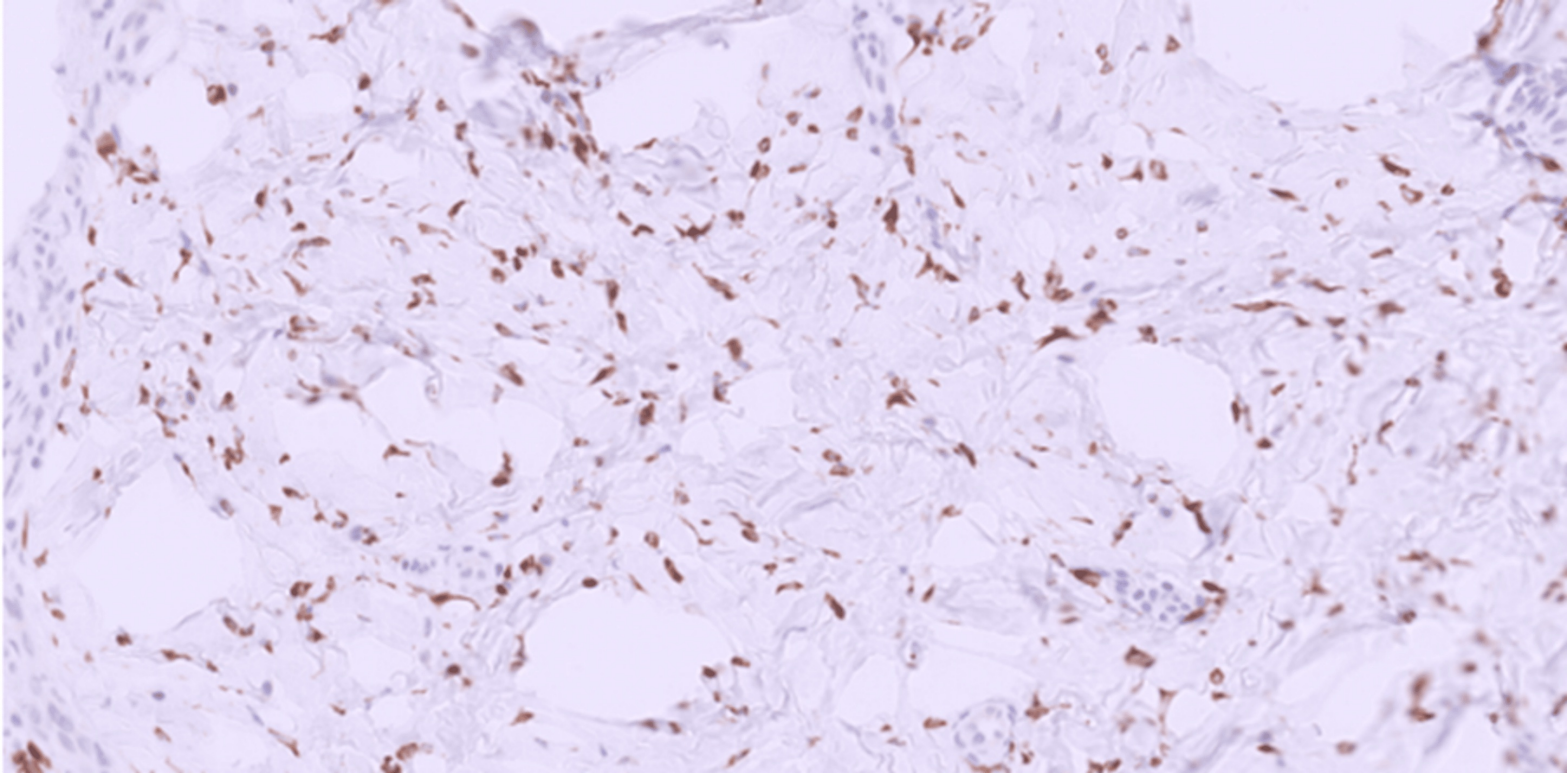
MUC4 immunostain. MUC4 immunostain was diffusely positive in the tumor cells (H&E, 20x).

**Figure 6:**
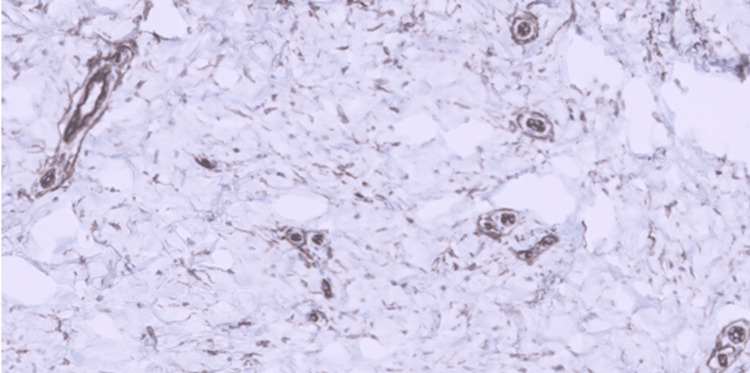
CD34 immunostain. CD34 immunostain demonstrated positive cytoplasmic staining within the tumor cells (H&E, 20x).

**Figure 7:**
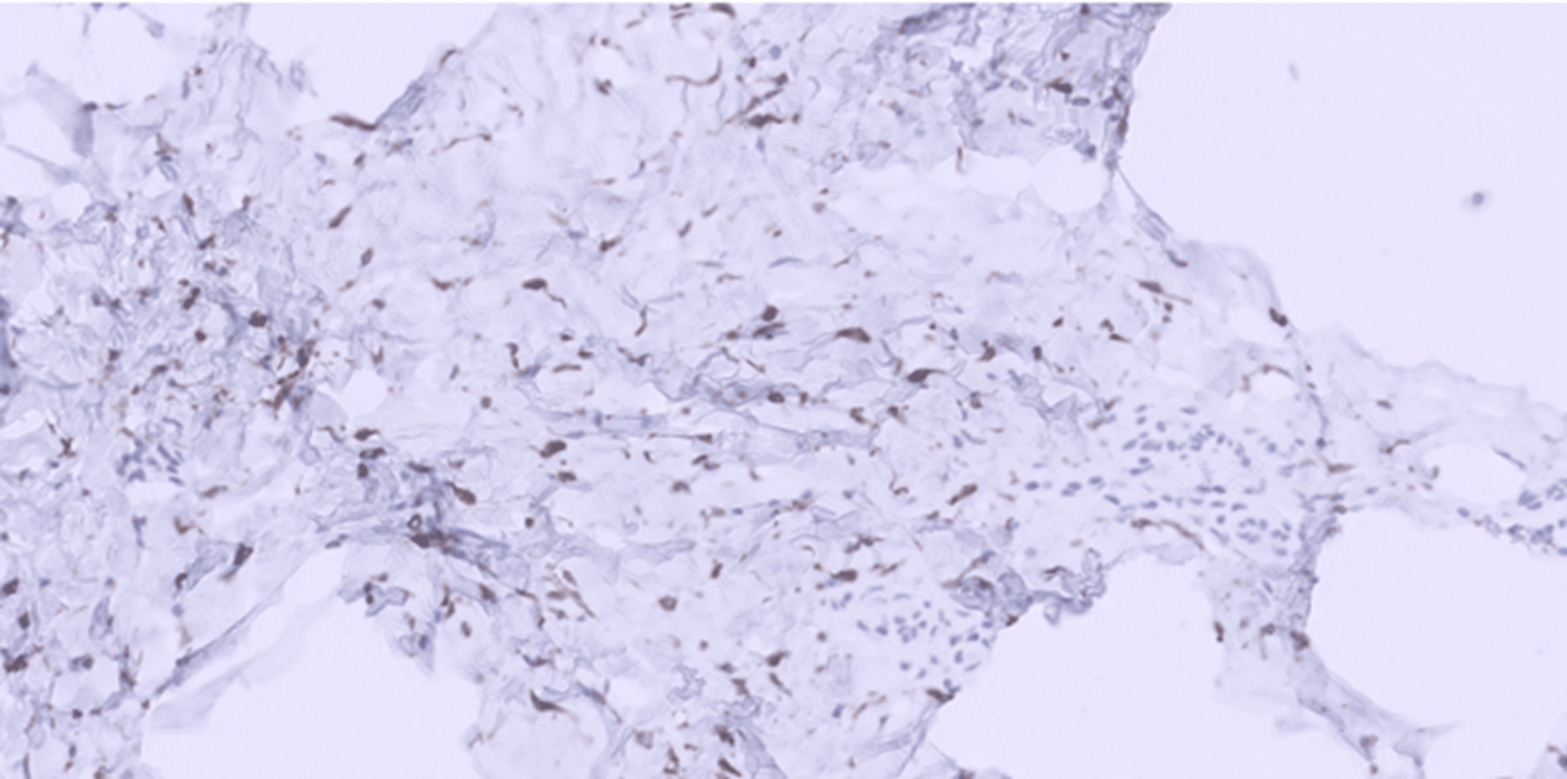
Desmin immunostain. Desmin immunostain showing cytoplasmic positivity within the tumor cells (H&E, 20x).

Nucleoli were inconspicuous, and no atypical mitotic figures or tumor necrosis were identified. The tumor showed no infiltrative growth into surrounding tissues. Immunohistochemical staining demonstrated diffuse positivity for MUC4 (Figure 5), CD34 (Figure 6), and desmin (Figure 7).

**Figure 8:**
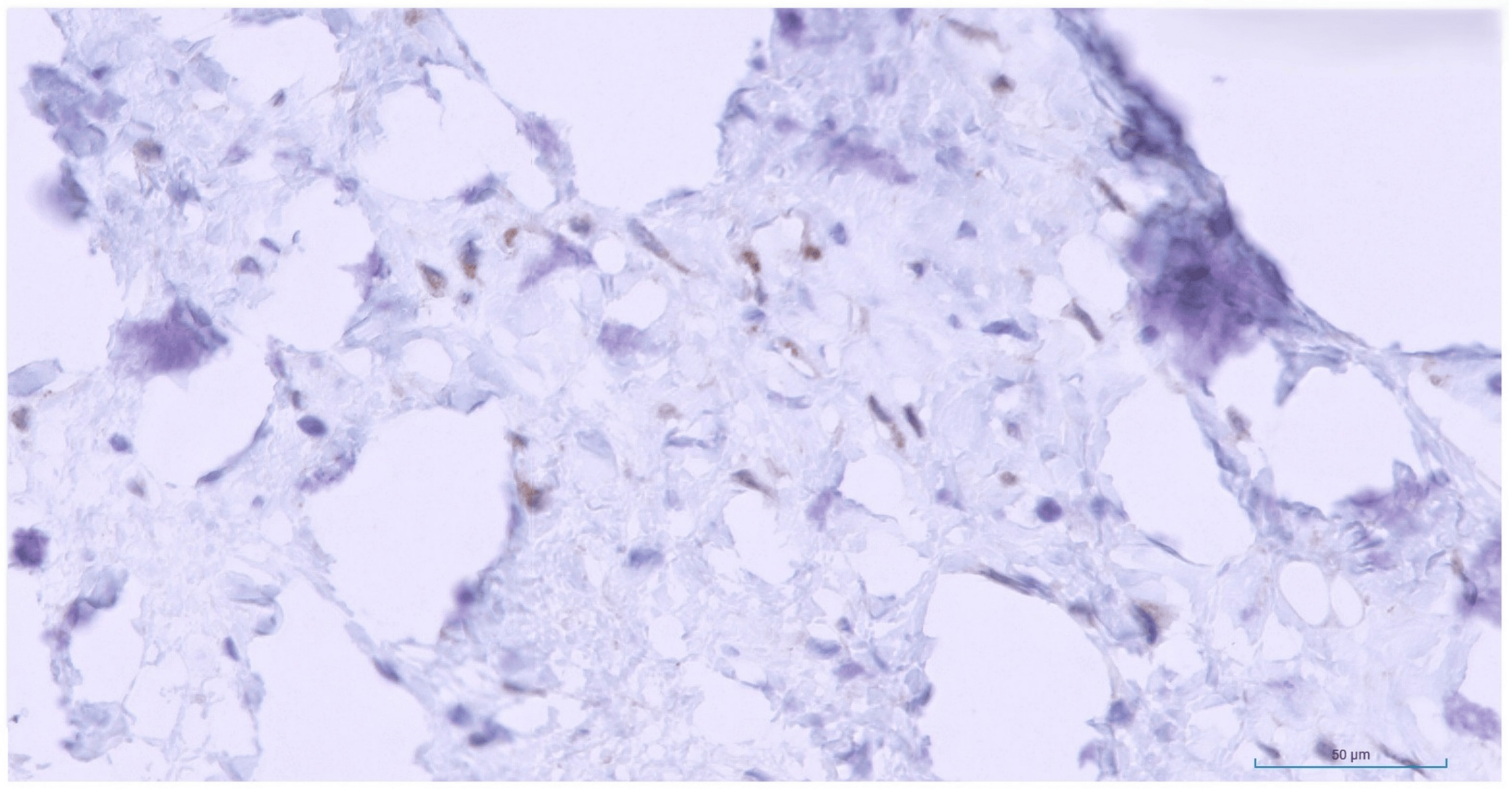
Beta-catenin immunostain. Beta-catenin immunostain demonstrating focal patchy cytoplasmic staining that is best regarded as negative (H&E, 40x).

Beta-catenin immunohistochemical stain demonstrated focal patchy cytoplasmic staining and was regarded as negative (Figure 8).

Smooth muscle actin, S100, and STAT6 immunohistochemical stains were negative. Fluorescence in situ hybridization for *MDM2* gene amplification and RNA fusion panel (next-generation sequencing-based assay that exploits reverse transcription of extracted RNA) were both negative. Tumor hotspot mutation panel (next-generation sequencing-DNA-based assay covering mutations in 50 genes) detected an inactivating *APC* mutation (p.K736*c.2206A>T). Subsequently, comprehensive next-generation sequencing (DNA-based assay that covers genetic alterations in 547 genes) demonstrated the same inactivating *APC* mutation with no additional significant variants seen, low mutation burden, and no apparent copy number alterations. The patient is still undergoing medical surveillance post-excision. 

## Discussion

In the original description of MUC4-positive fibroblastoma by Repetto et al. [1], tumors occurred in adult patients with a median age of 40 years; were predominantly deep; arose in the extremities, trunk, and retroperitoneum; displayed bland fibroblastic morphology with haphazard arrangement within a collagenous stroma; and showed diffuse co-expression of MUC4 and nuclear β-catenin in association with *APC *inactivation. The tumor reported here shares several features with this original series, including presentation in an adult patient, deep location within the extremity, bland fibroblastic haphazard collagenous morphology, diffuse MUC4 expression, and *APC* inactivation. However, β-catenin immunostaining revealed cytoplasmic rather than nuclear localization despite *APC* loss, which commonly disrupts the Wnt/β-catenin pathway by preventing β-catenin degradation, leading to nuclear accumulation and activation of target genes that promote tumor growth [4,5]. *APC* normally acts as a scaffold in the β-catenin destruction complex, targeting β-catenin for proteasomal degradation. When APC is mutated or lost, this complex fails, allowing β-catenin to accumulate in the nucleus.

The cytoplasmic localization of β-catenin in our patient, who has no known history of familial adenomatous polyposis, suggests that the downstream effects of *APC* inactivation may vary among fibroblastic tumors. Potential explanations include partial preservation of β-catenin regulatory control by certain *APC* truncations, altered nuclear transport, differences in fibroblastic differentiation, or involvement of noncanonical Wnt signaling pathways [6-8]. Additional cases will be required to determine whether cytoplasmic β-catenin represents a recurrent feature or a variant pattern within this tumor type.

The location of this tumor in the soft tissues of the hand introduces several additional diagnostic considerations, including LGFMS, fibroma of the tendon sheath, and palmar fibromatosis, all of which may present as well-circumscribed or multinodular spindle cell lesions in acral sites. LGFMS may arise in the upper extremity and demonstrates deceptively bland histology. Both LGFMS and MUC4-positive fibroblastoma may show hyalinized stroma, haphazard architecture, and low mitotic activity. However, LGFMS is characterized by alternating fibrous and myxoid zones, whorled or swirling growth patterns, and curvilinear vessels, none of which were identified here. LGFMS is also strongly associated with recurrent gene fusions, most commonly FUS::CREB3L2, or less frequently FUS::CREB3L1 and EWSR1::CREB3L1, which were absent in this lesion. The lack of a fibromyxoid component, absence of characteristic fusions, and overall simple architecture argue against LGFMS.

Fibroma of the tendon sheath, common in the hand, presents as a firm, small, slowly growing nodule attached to a tendon. Histologically, it exhibits a densely collagenized stroma with short bundles or stellate spindle cells, sometimes with slit-like vascular spaces and lobulated architecture. Unlike the current tumor, fibroma of the tendon sheath lacks diffuse MUC4 expression and is occasionally associated with USP6 rearrangements [9]. The combination of uniform cellularity, collagenous stroma, and circumscription may suggest fibroma of the tendon sheath, but the diffuse MUC4 labeling in this case distinguishes it.

Palmar fibromatosis, another common fibroblastic proliferation of the hand, arises in the superficial palmar fascia and can display a range of cellularity, from early plump myofibroblasts to dense, hyalinized spindle cell fascicles. Palmar fibromatosis may exhibit nuclear β-catenin positivity due to Wnt pathway alterations similar to desmoid-type fibromatosis [10]. In contrast, the current tumor showed cytoplasmic β-catenin and diffuse MUC4 positivity, making palmar fibromatosis unlikely.

Given the hand’s rich repertoire of fibroblastic and myofibroblastic proliferations with overlapping bland cytology and collagenous stroma, integration of morphology, immunohistochemistry, and molecular testing is essential. In this case, no pathogenic variants in *CTNNB1* were detected, and RNA fusion testing did not reveal rearrangements involving FUS::CREB3L2, FUS::CREB3L1, EWSR1::CREB family genes, or other fibroblastic tumor-associated fusions. The absence of these fusions, combined with diffuse MUC4 expression and *APC* inactivation, supports classification as MUC4-positive fibroblastoma.

Features that may prompt consideration of MUC4-positive fibroblastoma and subsequent molecular confirmation include a deep, relatively well-circumscribed, hypocellular spindle cell proliferation of bland fibroblasts arranged haphazardly within a collagenous stroma; absence of myxoid change, cytologic atypia, mitotic activity, or necrosis; and diffuse MUC4 immunolabeling. Recognition of these characteristics may aid pathologists in distinguishing this rare tumor from morphologically similar entities and guide appropriate molecular testing for *APC* alterations.

## Conclusions

This case, to our knowledge, represents the second documented example of MUC4-positive fibroblastoma and the first demonstrating cytoplasmic rather than nuclear β-catenin expression in the setting of APC inactivation. Our findings broaden current knowledge of the immunophenotypic spectrum of this rare and emerging entity.

Additionally, while the index report on this entity describes an indolent behavior with no established local recurrences or metastases to date, given the low number of reported cases thus far, the biologic potential of such tumors remains to be determined. Additional reports with detailed morphologic, immunohistochemical, and molecular correlation will be essential for establishing diagnostic criteria and clarifying biological behavior.
